# Cardiac Steatosis in HIV-A Marker or Mediator of Disease?

**DOI:** 10.3389/fendo.2018.00529

**Published:** 2018-10-11

**Authors:** Morgan Jacob, Cameron J. Holloway

**Affiliations:** ^1^St. Vincent's Hospital, Darlinghurst, NSW, Australia; ^2^University of Notre Dame, Darlinghurst, NSW, Australia; ^3^St.Vincent's Clinical School, University of New South Wales, Sydney, NSW, Australia; ^4^Victor Chang Cardiac Research Institute, Darlinghurst, NSW, Australia

**Keywords:** HIV, cardiac steatosis, pericardial fat, cardiovascular disease, metabolic dysregulation, systemic inflammation

## Abstract

Although people living with HIV (PLHIV) are approaching normal life expectancy, a limitation to achieving this goal is managing the higher prevalence of co-morbidities, including cardiovascular disease. Whilst ischaemic heart disease likely contributes to a large proportion of cardiac disease in the modern era of treatment, cardio-metabolic disease, including cardiac steatosis, akin to obesity-related heart disease, is also a possible mechanism of increased cardiac morbidity and mortality. HIV and other metabolic and inflammatory diseases affecting the heart, including obesity, share many cardio-metabolic abnormalities, with increased pericardial and myocardial fat content, in association with chronic systemic inflammatory changes and alterations in cardiac metabolism. Understanding the mechanisms of HIV-associated cardiac steatosis remains an important challenge, as managing the untreated metabolic and inflammatory precipitants may substantially improve cardiac outcomes for PLHIV.

## Introduction

Since the introduction of combined antiretroviral therapy (cART), HIV infection is recognized as a chronic manageable systemic condition. Cardiovascular Disease (CVD) remains a leading cause of death among PLHIV surviving more than 10 years after commencing cART (1). With the introduction of highly effective antiretroviral therapies, all-cause mortality in PLHIV decreased, however, CVD mortality has increased and is expected to continue to rise as cART availability expands to low-income countries (2). The exact nature of cardiac disease in the modern era of PLHIV has not been well established and this review considers the association of abnormal cardiac fat metabolism and deposition as a potential contributor to the increased cardiac morbidity and mortality seen in PLHIV.

## Cardiac disease in PLHIV

Poorly controlled HIV with the development of acquired immunodeficiency syndrome (AIDS) was associated with the development of pericardial diseases and cardiomyopathy, both of which were linked to reduced survival (3, 4). Heart failure in those with untreated HIV typically presented with left ventricular dilation and systolic dysfunction, with a median survival of 101 days after diagnosis (5). The incidence of HIV associated cardiomyopathy has significantly declined since the introduction of cART (4) and a modern cohort of PLHIV are more likely to suffer from ischaemic heart disease, myocarditis and the cardiac complications of systemic inflammatory and metabolic derangement (6, 7). Whilst the incidence of heart failure in PLHIV has overall reduced, heart failure remains more prevalent in PLHIV compared to negative controls. In a 2017 cohort study of 98,015, veterans living with HIV were at increased risk of heart failure with preserved ejection fraction, borderline heart failure with preserved ejection fraction and heart failure with reduced ejection fraction, with hazard ratio of 1.21, 1.37, and 1.61, respectively. Heart failure with preserved ejection fraction accounts for the greatest proportion of cases (8). The mechanism underlying the ongoing elevated incidence of heart failure in PLHIV using highly effective ART remains unknown, though it is likely multifactorial and partly related to abnormalities in fat metabolism.

Whilst HIV has been associated with all forms of cardiac disease, the focus has more recently shifted toward prevention of ischaemic heart disease. Males on cART, by the age 60, have a cumulative CVD incidence estimated at 20.5% compared to 14.6% in HIV-negative high-risk persons, and 12.8% in the US general population (9). Hence, HIV remains an important CVD risk factor in PLHIV using highly effective ART.

## Cardiac steatosis in PLHIV

**Figure 1:**
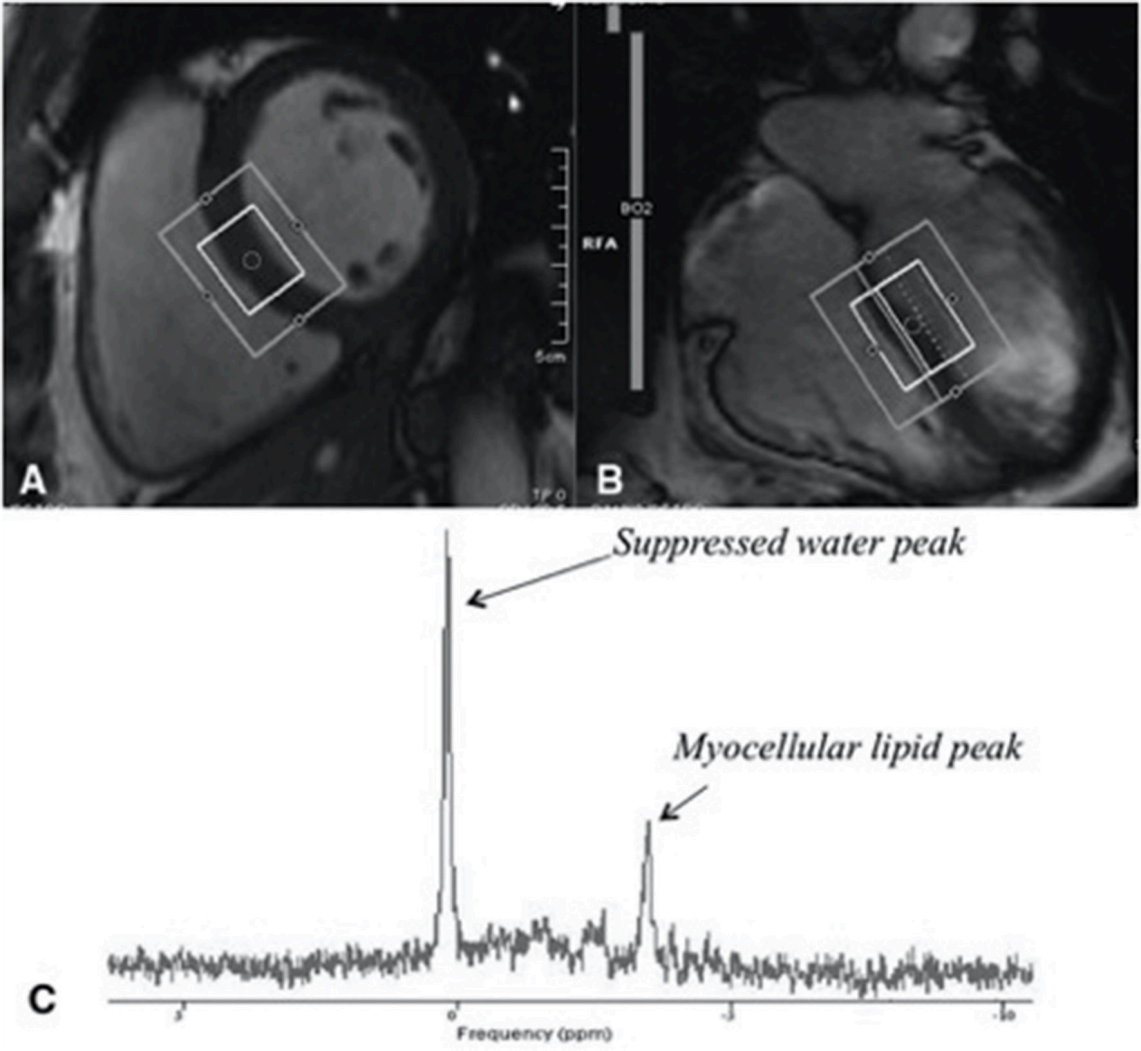
Assessment of myocardial steatosis using cardiac proton magnetic resonance spectroscopy (H1 MRS), a reliable, non-invasive means of quantifying myocardial lipids. **(A)** Short-axis image of the left ventricle with a voxel (box) in the interventricular septum of the heart of a midventricular slice (inner box). **(B)** Four-chamber image of the heart showing where the slice of the heart was selection. **(C)** An example of a proton MRS resonance spectrum, from a subject with HIV with identified myocellular lipid peak used in quantifying myocardial lipid. Figure taken from Holloway et al. (7).

Abnormalities in cardiac fat metabolism have been described in obese individuals, including increased epicardial and myocardial fat (10). The presence of visceral adipose tissue (VAT) is the best predictor of pericardial fat in non-diabetic abdominally obese subjects (11). Increased pericardial fat content is not exclusive to obese individuals and has been found in PLHIV. This was demonstrated in a large cohort study of 579 men with HIV and 353 men without HIV aged 40–70 years old, where epicardial adipose tissue (EAT) volume was assessed non-invasively via computed tomography. Men with HIV were found to have increased EAT compared to controls (*p* = 0.001), independent of other measures of adiposity including visceral adipose tissue and body mass index. Among men with HIV, after adjustment for age, race and cardiovascular risk factors, EAT volume was associated with the duration of combination ART. The duration of use of reverse transcriptase inhibitor azidothymidine (AZT) was associated with higher EAT volume than other ART. No associations were demonstrated between duration of use of either protease inhibitors, stavudine (d4T), or abacavir (ABC) therapy, and EAT volume (12). Factors found to be independently associated with raised EAT volume in PLHIV included age, male sex, visceral adipose tissue, waist circumference, total cholesterol and cumulative exposure to ART (13). Myocardial steatosis refers to raised adipose tissue within the myocardium, which can be measured using cardiac proton magnetic resonance spectroscopy (MRS), a non-invasive means of quantifying myocardial lipids by measuring their unique resonance frequency when passed through a magnetic field (14). Magnetic resonance imaging (MRI) and magnetic resonance spectroscopy (MRS) techniques allow the determination of cardiac function, fibrosis, and fat metabolism. Cardiac MRI allows determination of cardiac function, using cine images, and fibrosis, using Gadolinium contrast. Cardiac MRS is a technique that determines the chemical composition of the heart and can determine, for example, the fat content of the heart (Supplementary Figure 1). These techniques were used in 129 participants and compared with age-matched control subjects. Participants living with HIV undergoing cART (*n* = 90; 69.77%) had 47% higher median myocardial lipid levels (*P* < 0.003, Figure 1) and 74% higher median plasma triglyceride levels (both *P* < 0.001) (7). The association between PLHIV on ART and myocardial fat remained after adjustment for potential known confounders. The presence of cardiac steatosis was associated with elevated serum lipid level in subjects with HIV. However, positron emission tomography has demonstrated no changes in myocardial fatty acid uptake, esterification, and utilization in PLHIV compared with control subjects (15). Whilst an important technique to provide insights into disease, MRS is not yet a clinical tool and we do not advocate the use of cardiac MR or PET scans for the routine assessment of asymptomatic subjects with HIV.

HIV infection is linked to dysfunction in fat and glucose metabolism independent of ART (16). However, ART has also been shown to contribute to the metabolic dysfunction in PLHIV. Early ART regimen, in particularly protease inhibitors, are associated with visceral lipohypertrophy, dyslipidaemia and insulin resistance (17). Comparative studies between older and newer ART regimens have demonstrated improved metabolic profile with the use of newer ART regimen (18). However, long-term exposure to antiretroviral medications remains a metabolic risk for PLHIV. Among HIV-infected men, after adjustment for age, race and cardiovascular risk factors, epicardial adipose tissue volume was associated with the duration of combined ART (12). ART regimen including darunavir and atazanavir, which have been recommended by the Department of Health and Human Services (DHHS) for their favorable impact on glucose and lipid metabolism, have been associated with significant gain in limb fat, subcutaneous fat, visceral fat and trunk fat (19). Peripheral metabolic complications in other diseases, such as diabetes mellitus and obesity, are associated with cardiac functional derangements, morbidity, and mortality, which are independent of blood pressure, body mass index, and coronary disease. Whether lipid and glucose abnormalities predispose to cardiac metabolic alterations and deposition of pericardial and myocardial fat in PLHIV remains unknown.

## Cardiac steatosis, as an independent risk factor for CVD

The question remains whether the relationship between pericardial fat and cardiovascular disease is merely a marker or mediator of risk. Recent data from the Multi-Ethnic Study of Atherosclerosis, a prospective study with a median 12.2-year follow-up of 4,234 participants found that in the general population pericardial fat is associated with elevated rates of CVD events, and increase left ventricular mass (20). There is a clear relationship between pericardial fat and coronary artherosclerosis. In PLHIV, The Multicentre AIDS Cohort Study demonstrated that pericardial fat was associated with coronary atherosclerosis. Among men with coronary artery calcium (CAC) epicardial adipose tissue was associated with the extent of CAC (*p* = 0.006) (12). Epicardial fat share the same microcirculation as the myocardium, highlighting their essential functional relationship (21). The Framingham Heart Study found that pericardial fat but not intrathoracic fat was associated with coronary artery calcification (22). The location of fat deposits is therefore important in determining the impact on the vasculature, further supporting the association between pericardial steatosis and CVD (20). Recent studies have highlighted the pro-inflammatory nature of pathological fat deposits. Obese models have demonstrated a transformation of perivascular adipose tissue into a pro-inflammatory phenotype with the upregulation of proinflammatory cytokines (23). In virologically suppressed PLHIV on ART, perivascular fat is associated with biomarkers of inflammation, insulin resistance, and subclinical atherosclerosis (24). The proximity of pericardial fat combined with a pathological switch into a pro-inflammatory state is believed to create an inflammatory milieu promoting coronary artery disease in PLHIV.

A cross-sectional analysis performed in obese subjects identified that increasing myocardial triglyceride content on MRS remained correlated to diastolic dysfunction after controlling for LV mass, systolic blood pressure, and insulin resistance (10). The term cardiac lipotoxicity refers to fatty-acid sequestration and oxidation and is associated with a build-up of toxic fatty-acid metabolites within cardiomyocytes, leading to cardiomyopathy (25). Cardiac lipotoxicity may underline the asymptomatic myocardial dysfunction seen in PLHIV. However further studies are needed to investigate this association. Increased rates of fatty acid oxidation found in obese models impairs glucose oxidation (26). Impaired glucose metabolism in cardiac steatosis is widely held as an important factor in cardiac dysfunction. Cardiac-specific deletion of pyruvate dehydrogenase, a rate limiting enzyme involved in glucose oxygenation, results in impaired glucose oxidation rates and diastolic dysfunction (27). Cardiac lipotoxicity and impaired glucose metabolism may explain cardiac dysfunction found in PLHIV, which predominantly presents with diastolic dysfunction (8). However further studies in PLHIV are needed to investigate this association.

Weight loss, in obese subjects, has been shown to improve impaired cardiac energetics and myocardial relaxation, thus improving diastolic dysfunction (28). An improvement in cardiac function associated with the reduction in cardiac steatosis would support an aetiological link between cardiac steatosis and systolic and diastolic dysfunction observed in PLHIV.

## Inflammatory changes causing steatosis

Immunological derangements in both the innate and adaptive immune system have been described in PLHIV. The Human Immunodeficiency Virus primarily targets the adaptive immune system resulting in CD4+ T cell depletion and CD8+ T-cell activation (29). The resulting derangement in the adaptive immune system, marked by a high absolute CD8+ T-cell count as well as a lower CD4: CD8 ratio, have been associated with an increased risk of myocardial infarction among HIV-infected individuals (30). In addition to T cell changes the phenotype of macrophages is affected in PLHIV. Soluble CD14, a non-specific marker of monocyte activation, a key cell in the innate immune system and atherogenesis, has been associated with calcified coronary plaque among PLHIV (31). The frequency of patrolling non-classical monocytes CD14+CD16+ was elevated among PLHIV presenting with an acute coronary syndrome and also persisted among those with undetectable viral load (32). These CD14+CD16+ monocytes subsets exhibit properties that promote atherogenesis, including a higher affinity for vascular surfaces and migration into atherosclerotic lesions (33). The observed increased level of CD14+CD16+ monocytes subsets may contribute to the elevated cardiovascular risk found in PLHIV.

Despite viral suppression, PLHIV have evidence of chronic systemic inflammation as demonstrated by elevated inflammatory markers, which may underlie the increased risk of ischaemic heart disease and cardiometabolic abnormalities (34). Elevated C- reactive peptide (CRP), a non-specific acute phase protein marker of inflammation, for instance, has been associated with increased coronary events (35). A retrospective analysis looking at PLHIV found that the presence of HIV and an elevated CRP have a synergistic effect on acute myocardial infarction (AMI) risk, with a hazard ratio of 4.11. Increased CRP and HIV infection individually were each associated with an approximately 2-fold increased risk for AMI compared to those with neither risk factors (36). Non-HIV conditions with systemic inflammation, such as systemic lupus erythematosus, rheumatoid arthritis and diabetes mellitus, are associated with increased pericardial fat and cardiac steatosis (37–39). Further studies into the relationship of cardiac fat and inflammatory conditions may show that inflammation is a shared pathway in the development of pathologic heart fat.

## Therapeutics targets to reduce CVD risk in PLHIV

There is likely a complex interplay between untreated inflammation and ongoing metabolic alterations leading to fat deposition in PLHIV, likely translating to a difficulty in finding a simple solution to reducing pericardial and myocardial cardiac fat deposition. Given the higher proportion of traditional modifiable risk factors in PLHIV, including; smoking, hypertension, diabetes and dyslipidaemia, modification of heart disease risk should start with these factors (40). Weight loss in obese patients is associated with the reduction of cardiac steatosis and improvement in cardiac function (28, 41), therefore, aiming for a normal BMI is recommended. Medications aimed at reducing cardiac steatosis is a potential therapeutic target for the treatment of cardiac dysfunction in PLHIV.

Through decades of extensive research, heart disease in patients with diabetes and obesity are understood as the result of alterations in glucose and fat metabolism and inflammation, leading to vascular dysfunction, structural, and metabolic heart disease. We propose the same mechanisms may be implicated in PLHIV and we can therefore learn from treatment used in similar metabolic diseases to improve fat metabolism. Hence, the next logical step would be to alter factors which lead to changes in glucose and fat metabolism, which may alter pericardial and myocardial fat deposition, in addition to reducing vascular risk.

The emergence of newer diabetic medications, such as glucagon-like peptide-1 (GLP-1) agonists and sodium/glucose co-transporter (SGLT)2 inhibitors confer benefits on cardiovascular outcomes in patients with diabetes (42, 43). Perhaps these medications will confer such benefits in non-diabetic PLHIV in whom impaired glucose metabolism is believed to be an important factor in the development of CVD.

Statins have become an important part of both primary and secondary CVD prevention (44) In the SATURN trial, the use of rosuvastatin and atorvastatin at their maximal dose in individuals with angiographic evidence of CAD was associated significant regression of coronary arthrosclerosis (45). Likewise, statins are believed to have an important role in the prevention of CVD events in PLHIV. However, the evidence for the use of statins in PLHIV with otherwise low traditional risk factors for CVD remains unclear. The SATURN-HIV study, a 96-week double blind, randomized clinical trial, investigating the use rosuvastatin in PLHIV on ART with raised inflammatory markers, found significantly reduced low density lipid (LDL) levels and slower progression of carotid artery intima-media thickening in the treatment group (*n* = 72) compared to the placebo group (*n* = 75). This study, however, did not demonstrate significant change in coronary artery calcium (46). A randomized double-blind placebo controlled trial where 40 PLHIV with subclinical coronary atherosclerosis were randomized to 1 year of treatment with atorvastatin (*n* = 19) or placebo (*n* = 21) found that the treatment arm induced coronary plaque regression among participants living with HIV (47). In addition to a reduction in LDL levels, the use of rosuvastatin in PLHIV on ART is associated with a reduction in markers of inflammation as well as T cell and monocyte activation (48). Therefore, the cardioprotective action of statins in PLHIV is believed to occur at its effect on metabolism and the immune system. As discussed above, PLHIV are at a high risk of developing CVD with a cumulative CVD incidence at the age of 60 of 20.5% compared to 12.8% in the US general population (9). However, traditional CVD risk scales under estimate CVD disease risk in PLHIV. Therefore, it remains unclear if PLHIV with low CVD risk factors and 10-year ACC/AHA risk of <7.5% would benefit from treatment. The REPRIEVE trial, a randomized controlled trial assessing the effectiveness of pitavastatin in reducing primary cardiovascular events for patients on ART with 10-year ACC/AHA risk of <7.5%, is expected to give light on the use of statins as primary prevention for CVD in PLHIV with low CVD risk by the year 2020.

It is unknown whether reduced inflammation will lower cardiac fat deposition. A recent randomized controlled trial using the interleukin 1beta blocking agent, canakinumab, has been shown to decrease myocardial infarction in people at risk (49). In support of its use in PLHIV, Huse et al. demonstrated a reduction in inflammatory markers (IL-6, hsCRP) in 10 subjects with HIV treated with canakinumab (50). The use of canakinumab, however, was associated with higher incidence of fatal infection than the placebo group which may limit its use in PLHIV (49). The use of rosuvastatin in ART treated PLHIV was associated with a reduction in markers of inflammation as well as T cell and monocyte activation. There are several ongoing trials evaluating the effects of therapeutics including methotrexate, canakinumab, and probiotics for vasculature function and/or vascular inflammation, which could potentially translate to improve cardiac fat metabolism.

## Conclusion

The era of cART has been associated with a paradigm shift from cardiomyopathy to cardiometabolic and atherosclerotic disease in PLHIV; which are both likely sequelae of untreated chronic metabolic and inflammatory changes, from both the virus and cART. Despite viral suppression, PLHIV have evidence of chronic systemic inflammation. Most, if not all systemic inflammatory diseases, increase the risk of abnormal fat metabolism, myocardial infarction and heart failure, emphasizing the role of inflammation in the pathogenesis of heart disease. Therefore, the way to improve cardiac outcomes in PLHIV may be dependent on improving inflammatory and metabolic states. Cardiac steatosis in HIV is not simply age-related change and as such, a systemic approach to understanding fat deposition is required to improve HIV outcomes.

## Author contributions

MJ performed a literature review of the current evidence regarding cardiac steatosis within people living with HIV. MJ was involved in the planning and drafting of the article. CH was involved in the conception of the article. CH provided guidance as to relevant latest contributions in the field of cardiac steatosis. CH drafted the section on therapeutic targets as well as critical revision the article.

### Conflict of interest statement

CH has received unrestricted research funding from Bayer, travel grants from Gilead Sciences and honoraria from Bayer, ViiV, and Gilead. The remaining author declares that the research was conducted in the absence of any commercial or financial relationships that could be construed as a potential conflict of interest. The handling Editor declared a shared affiliation, though no other collaboration, with one of the authors CH.
