# Supplementary material for: Cardiac Steatosis in HIV-A Marker or Mediator of Disease?
Source: Front Endocrinol (Lausanne). 2018 Oct 11;9:529. doi: 10.3389/fendo.2018.00529 (PMC6193415; doi:10.3389/fendo.2018.00529)
Supplement: Supplementary file 1 [file Image_1.pdf]

Supplementary Material

Morgan Jacob, Cameron J Holloway*

*** Correspondence:** A/Prof Cameron Holloway: cholloway@svha.org.au

Supplementary Figures

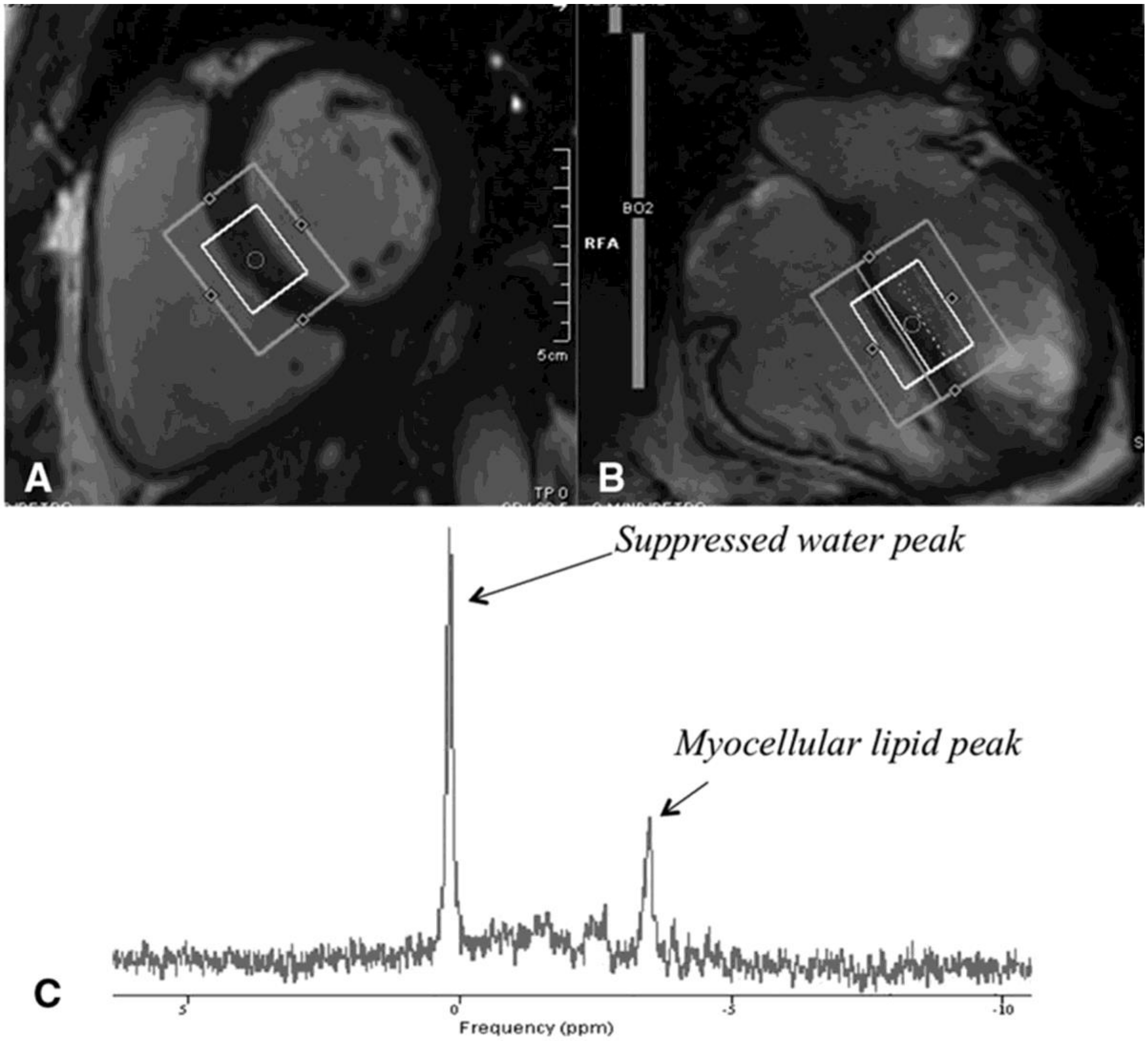

Supplementary Figure 1. Assessment of myocardial steatosis using cardiac proton magnetic resonance spectroscopy (H1 MRS), a reliable, non-invasive means of quantifying myocardial lipids. **A**, Short-axis image of the left ventricle with a voxel (box) in the interventricular septum of the heart of a midventricular slice (inner box). **B**, Four-chamber image of the heart showing where the slice of the heart was selection. **C**, An example

of a protonMRS resonance spectrum, from a subject with HIV with identified myocellular lipid peak used in quantifying myocardial lipid. Figure taken from *Circulation*. 2013;128:814-822, with permission.
